# Effects of the Liverpool Citizens Support Scheme on mental health-related service utilisation: evidence from a natural experiment using instrumental variable analysis

**DOI:** 10.1186/s12889-026-27681-x

**Published:** 2026-05-11

**Authors:** Huihui Song, Emma Coombes, Harris Kaloudis, Benjamin Barr

**Affiliations:** 1 University of Liverpool, Liverpool, UK; 2 Lancaster University, Lancaster, UK

**Keywords:** Local welfare reform, Mental health, Panel data, Instrumental variable, Social policy

## Abstract

**Background:**

There is limited evidence on how local welfare schemes may affect mental health. This study estimated the impact of the Liverpool Citizens Support Scheme on mental health-related service utilisation in one of the most socioeconomically disadvantaged areas of the UK. Introduced in 2013, the Liverpool Citizens Support Scheme aims to alleviate acute financial hardship by providing small grants to households in financial crisis. The scheme was open to all residents meeting financial eligibility criteria, but a policy change implemented in April 2023 restricted access for individuals living in social housing. This policy change constitutes a natural experiment that provides an opportunity to assess the mental health impact of local welfare provision.

**Data and methods:**

We used quarterly administrative data from the Liverpool Citizens Support Scheme from 2018 to 2023, linked at the small-area-level to healthcare data. The primary outcome was a quarterly mental health index constructed using four indicators of mental health service use: antidepressant prescriptions, mental health-related general practitioner (GP) consultations, accident and emergency (A&E) visits, and emergency hospital admissions. To address potential endogeneity in scheme uptake, we used an instrumental variable defined as the interaction between a post-policy indicator and the proportion of social housing tenants living in each neighbourhood, and estimated the association between grant provision and mental health-related service utilisation using two-stage least squares regression.

**Results:**

Each additional £1 in grants issued per person per quarter was associated with a -0.07 reduction in the mental health index (95% CI: -0.11 to -0.02; *p* < 0.01), and with reductions of 309 antidepressant prescriptions per 1,000 of the population (95% CI: -536 to -83), 2.52 GP consultations per 1,000 (95% CI: -4.88 to -0.15), and 0.61 A&E visits per 1,000 (95% CI: -1.11 to -0.11). Under the assumptions of the instrumental variable design, these associations would correspond to an estimated saving of £0.45 to the health service for every £1 in grants issued (95% CI: £0.07 to £0.82), and £7,394,755 in total between 2018 and 2023 (95% CI: £1,150,295 to £13,474,887). Subgroup analysis suggested that differences in service utilisation were more pronounced for affected males, while there was no evidence of differential impact across age groups or income levels.

**Conclusions:**

These findings are consistent with the interpretation that welfare support delivered through the Liverpool Citizens Support Scheme may be associated with reduced demand for mental health-related healthcare services. The study points to the potential value of local welfare interventions in supporting population health and addressing health inequalities in deprived communities, and suggests how such schemes may contribute to considerable cost savings to health services whilst also alleviating financial hardship.

**Supplementary Information:**

The online version contains supplementary material available at 10.1186/s12889-026-27681-x.

## Introduction

Mental health conditions have become a pressing public health concern in the UK, with one in four adults affected annually and 17% of adults living with common conditions such as anxiety or depression [1]. Mental disorders are now among the leading causes of ill health and disability, costing the UK economy an estimated £300 billion per year [2].

Welfare policy reforms have emerged as a key structural determinant of mental health outcomes. For example, more generous welfare provisions have been associated with improvements in mental health [3], while the introduction of a new welfare programme in the UK - Universal Credit has consistently been linked to worsening mental health [4–7]. In response to national reforms and increased devolution of national programmes, local welfare schemes have expanded across the UK. Local welfare schemes are discretionary programmes that are provided or commissioned by local authorities and offer cash or in-kind support, accompanied by welfare advice, to individuals experiencing financial crises. Although local welfare schemes play a vital role in addressing gaps in national welfare provision, they remain understudied [8]. There is therefore a need to better understand how local welfare schemes may address local needs, while also recognising that overstretched welfare support systems may risk reinforcing stigma and inequalities [9].

Liverpool provides a particularly relevant case study for examining the role of local welfare schemes. It is one of the UK’s most socioeconomically deprived cities and it experiences disproportionately high levels of mental ill health, with 22% of adults in the Liverpool City Region affected, which is well above the national average [10]. In response, Liverpool City Council invests around £6 million annually in local welfare schemes, which exceeds the commitment of many other local authorities. This makes the region a critical site for understanding how local welfare schemes function.

Among local welfare scheme initiatives, the Liverpool Citizens Support Scheme stands out as one of the largest and most comprehensive. Introduced in 2013, the Liverpool Citizens Support Scheme aims to address acute financial hardship, particularly in the wake of austerity measures and the post-pandemic cost-of-living crisis. The scheme comprises two main components. Home Needs Awards support individuals and families in setting up or maintaining a home by providing essential household items, including furniture, white goods, domestic appliances, bedding, and crockery. This award is particularly targeted at people moving into independent accommodation after leaving care or prison, or those forced to relocate due to domestic violence or other emergency circumstances. Urgent Needs Awards offer short-term financial assistance to cover basic living necessities such as food, fuel, essential clothing, and items for children, as well as support for those affected by sudden crises such as fires or floods. Liverpool Citizens Support Scheme clients are limited to two awards in a 12-month period, although Liverpool City Council has discretion to make additional awards where they feel it is appropriate to do so. Between 2018 and 2023, the scheme supported 87,746 applications and distributed a total of £16.4 million in financial assistance (an average of £187 per beneficiary), serving as a critical safety net for those most affected by economic adversity.

**Fig. 1:**
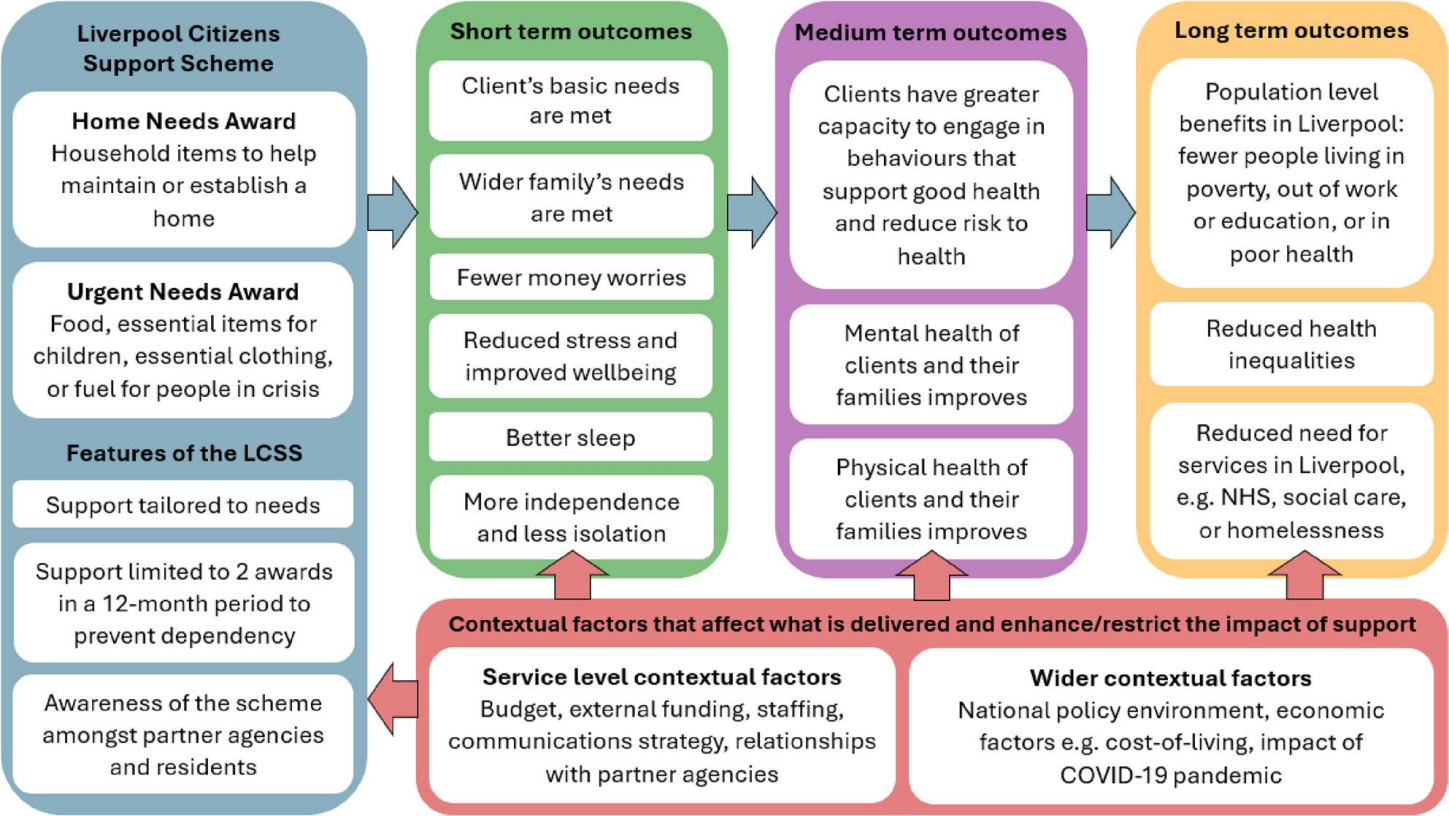
Logic model highlighting pathways through which the Liverpool Citizens Support Scheme is expected to impact mental health

The potential for the Liverpool Citizens Support Scheme to influence mental health-related service utilisation is both immediate and long-term as shown in the logic model in Fig. 1. In the short-term, the provision of financial or in-kind support can directly alleviate hardship and reduce psychological distress. Longer term, resolving acute financial crises may yield sustained mental health benefits, both at an individual level for clients of the service and also for the wider healthcare system by reducing demand for clinical interventions.

A significant policy reform in April 2023 introduced a structural change to the Liverpool Citizens Support Scheme by limiting access for individuals living in social housing (see further detail below). This policy created an exogenous variation in access to the Liverpool Citizens Support Scheme that particularly affected a highly vulnerable population. As such, it offers a unique opportunity to evaluate the impact of Liverpool Citizens Support Scheme on mental health-related service utilisation.

Despite significant investment in local welfare schemes, empirical evidence on their health impacts remains limited. While qualitative studies have offered insights into how such schemes shape lived experiences [9, 11, 12], quantitative evaluations are comparatively scarce [13]. Much of the existing research has focused on the delivery of welfare advice in healthcare or community settings, often constrained by small sample sizes [14, 15]. To date, one recent study offers encouraging evidence that providing welfare advice through health services can improve mental health by reducing financial insecurity [16]. However, such findings remain limited in scope.

Taken together, these gaps underscore the need for more rigorous, causal quantitative evaluations of local welfare schemes and their broader health impacts. Responding to this need, the present study applied an instrumental variable (IV) approach to estimate the causal effect of the Liverpool Citizens Support Scheme on mental health-related service utilisation, leveraging the 2023 policy reform as a source of exogenous variation.

## Methods

### Data, exposure, outcome, and control measures

We defined exposure to the Liverpool Citizens Support Scheme as the total value of grants issued per head of population within each age (15–34, 35–49, 50–64, and 65+) and gender group within each Lower Super Output Area (LSOA) and each quarter between 2018 and 2023. LSOAs are small geographic units used in the UK for statistical purposes, typically containing around 1,500 residents. Within our stratified panel, the average population per cell is approximately 175 individuals. These data were derived from the Liverpool Citizens Support Scheme administrative database provided by Liverpool City Council.

To examine the effect of Liverpool Citizens Support Scheme uptake on mental health, we derived quarterly mental health-related service utilisation for the same age and gender group within each LSOA for each quarter, using data provided by the National Health Service (NHS) [17], covering Liverpool’s entire population of approximately 497,000 individuals (see Appendix 1 for further details on data sources).

We selected four indicators of mental health-related service utilisation that represent complementary dimensions of mental health care for common mental disorders across primary and secondary services. This was consistent with prior UK studies, that had found these measures are sensitive to changes in welfare provision [16]. These indicators were calculated as rates per 1,000 population:

(1) The rate of antidepressant prescribing was calculated as the number of Average Daily Quantities (ADQs) of antidepressants prescribed by GP practices divided by the population. ADQs are a standardised measure of prescribing quantities used by the NHS in England [17]. All antidepressant prescriptions were included within the British National Formulary (BNF) Chap. 4.3, excluding Amitriptyline, which is now largely used for pain management.

(2) The rate of GP consultations for mental health conditions were calculated as the number of mental health-related GP consultations divided by the population. These were defined as contacts with a GP or nurse in general practice, coded on the GP clinical system with one or more of a set of mental health-related codes (see Appendix 1 for details of the code list).

(3) The rate of A&E attendances for mental health issues, computed as the number of mental health-related attendances at an accident and emergency department (A&E). The diagnostic codes used for these measures are in Appendix 1 [18].

(4) The rate of emergency hospital admissions for mental health. Diagnostic codes used for these measures are in Appendix 1 [19–22].

Although these indicators covered different diagnostic clusters, they collectively provided a comprehensive picture of mental health-related service use. To address potential inconsistencies and summarise the overall burden of mental health-related service utilisation at the population level, we applied factor analysis to the four indicators to capture the common variation across these measures and derived a composite index. The resulting index was standardised with a mean of zero and a standard deviation of one, so that a one-unit increase represents one standard deviation higher overall mental health service utilisation relative to other LSOAs in Liverpool. The index provided a measure of relative mental health-related service utilisation for each age and gender group within each LSOA in each quarter.

Other mental health-related measures, such as antipsychotic prescribing and referrals to psychological therapies, social prescribing, or psychiatry, were considered but excluded due to data availability, incomplete or inconsistent recording at the LSOA level across the study period, and because the incidence of psychotic disorders is unlikely to be sensitive to short-term changes in socioeconomic conditions [16].

This produced a quarterly panel dataset of policy exposure and outcomes between 2018 and 2023 for each age and gender group within each LSOA, giving a total of 57,216 observations. This dataset was supplemented with UK Census data to capture key area-level characteristics, including total population, the proportion of households in social rented housing, and the number of unemployed individuals at the LSOA level. Finally, the income score from the English Index of Multiple Deprivation 2019 was merged into the dataset. These variables were time-invariant over the study period. We included the unemployment rate per individual as a control variable, as well as the interaction between income score and time to capture changes in socioeconomic conditions over time.

### Policy changes

In April 2023, the Liverpool Citizens Support Scheme introduced a policy change that imposed expenditure limits on grants for tenants in social housing. This reform withdrew support for Home Needs Award provision for individuals living in social housing. Under this reform, Registered Social Landlords were expected to meet their tenants’ needs by providing essential household items, such as furniture or white goods. Landlords were then able to recover their costs by levying additional service charges, which tenants could in turn cover via their Universal Credit or Housing Benefit payments. We utilised this policy shift (hereafter referred to as the Liverpool Citizens Support Scheme Policy Control) to investigate the impact on mental health-related service utilisation at the LSOA level.

**Fig. 2:**
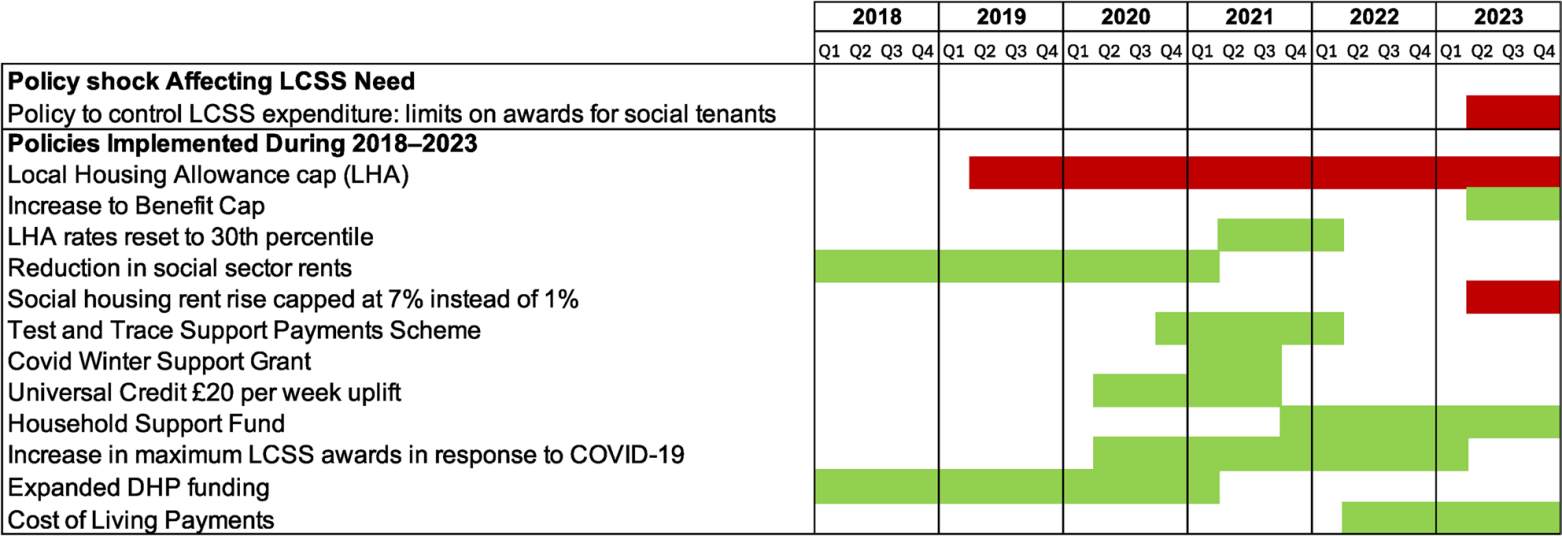
Timeline of policy changes between 2018 and 2023, where green denotes policies that were likely to have had a positive impact on residents’ finances and red denotes those that were likely to have had a negative impact

In addition to the Liverpool Citizens Support Scheme Policy Control described above, we included 12 other relevant policy changes at both national and local levels during the study period that could be confounders in our analysis. Figure 2 presents the timeline of these policy changes, with full details of each policy change provided in Appendix 2. Some policies potentially had a positive impact on residents’ finances (e.g. the increase to benefit cap, shown in green), whereas other policies potentially had a negative impact (e.g. the Local Housing Allowance cap, shown in red). These 12 policy changes, together with their interactions with the percentage of social housing tenants, were included as controls in the analysis, as they were likely to have had disproportionate impacts on social housing residents.

### Instrumental variable indicator

The Liverpool Citizens Support Scheme Policy Control variable, interacted with the proportion of social housing tenants at the LSOA level, was used as an instrumental variable to estimate the effect of Liverpool Citizens Support Scheme grant provision on mental health. An instrumental variable (IV) is a variable that influences the likelihood of individuals receiving an intervention (in this case the Liverpool Citizens Support Scheme), but does not influence the outcome (in this case mental health-related service utilisation), except through its effect on the intervention. In our study, we were assuming that the change in the policy, which had a greater impact on areas with higher levels of social housing after 2023, could only impact the change in mental health in those areas by reducing the probability of the population receiving the Liverpool Citizens Support Scheme. The interaction was selected as an “instrumental variable” therefore, on the basis that the policy disproportionately affected social housing tenants, generating variation over time in welfare access that is plausibly exogenous to change in local mental health-related service utilisation after 2023. Under this framework, the IV estimates identify a local average treatment effect (LATE), capturing the effect of grant provision among areas whose exposure was influenced by the policy-induced restriction.

### Statistical analysis

To estimate the impact of the Liverpool Citizens Support Scheme on clients’ mental health, we employed an IV approach to address potential endogeneity (i.e. that poor mental health might lead to increased Liverpool Citizens Support Scheme uptake), using the Policy Control variable interacted with the proportion of social housing tenants as outlined above. The first and second stages of the two-stage least squares (2SLS) model were specified as follows:

First stage:

$$\begin{aligned} \:{LCSS\:grant\:rate}_{it}\:=\:&{\alpha\:}_{0}+{\alpha\:}_{1}\left({Social}_{i}*{LCSS\:Policy\:control}_{t}\right)\\&+{\alpha\:}_{2}{Unemployment\:Rate}_{i}\\&+{\alpha\:}_{3}\:{Income\:Score}_{i}*\:{time}_{t}\\&+{\alpha\:}_{4}Remaining\:Policy*{Social}_{i}\\&+{{\uptau\:}}_{t}+{u}_{i}+{\epsilon\:}_{jt} \end{aligned}$$

Second stage:

$$\begin{aligned} \:{Mental\:health\_Var}_{it}=\:&{\sigma\:}_{0}+{\sigma\:}_{1}{\widehat{LCSS\:grant\:rate\:}}_{it}\\&+{\sigma\:}_{2}{Unemployment\:Rate}_{i}\\&+{\sigma\:}_{3}\:{Income\:Score}_{i}*\:{time}_{t}\\&+{\sigma\:}_{4}Remaining\:Policy*{Social}_{i}\\&+{{\uptau\:}}_{t}+{u}_{i}+{\epsilon\:}_{jt} \end{aligned}$$

In the first stage, we modelled our potentially endogenous policy exposure variable, the grant rate, as a function of our IV and a comprehensive set of controls to account for observable area-level differences that may confound the relationship between Liverpool Citizens Support Scheme uptake and mental health-related service utilisation. These included the local unemployment rate, serving as a proxy for economic hardship, and interactions between remaining national and local welfare policy indicators (including the contemporaneous 7% rent cap, as well as a further 11 policies shown in Fig. 2) and the local proportion of social tenants. $\:{{\uptau\:}}_{t}$ represents time fixed effects, and $\:{u}_{i}$ represents unit fixed effects (defined by LSOA, gender, and age group). The error term $\:{\epsilon\:}_{jt}$ captures the remaining unexplained variation.

In the second stage, we regressed the mental health-related healthcare services (comprising a composite index and four additional mental health indicators) on the predicted values of the Liverpool Citizens Support Scheme grant rate derived from the first stage. The coefficient $\:{\sigma\:}_{1}$ represents our primary parameter of interest, measuring the impact of Liverpool Citizens Support Scheme grant provision on mental health-related service utilisation. The same set of control variables and fixed effects were included to ensure consistency across both stages of the model.

We further conducted two sensitivity analyses to assess the robustness of our findings. First, the validity of the instrumental variable was tested using the first-stage F-test and the Wu–Hausman test in the second stage. Second, we performed a short-window analysis limited to the period 2021–2023 to minimise potential confounding effects of the COVID-19 pandemic. To further explore heterogeneity in the effects, subgroup analyses were conducted by gender, age group, and income level.

## Results

The panel dataset comprises 2,384 unique segments, defined by the cross-classification of 298 LSOAs in Liverpool, two gender categories (female and male), and four age groups (15–34, 35–49, 50–64, and 65+). The data spanned quarterly periods from 2018 to 2023, resulting in a total of 57,216 observations.

Table 1 presents summary statistics for the analytic sample, including key mental health-related healthcare services, the policy exposure variable, and relevant control variables. The table reports the mean and standard deviation for each variable.

**Table 1:** Summary statistics of quarterly data from 2018 to 2023

Variables	Full sample (*n* = 57,216)	
	Mean	SD
Outcome variables		
Quarterly ADQs of antidepressant prescribing per 1,000 people	18,554	13,263
Quarterly mental health-related GP consultations per 1,000 people	53	43
Quarterly mental health-related A&E attendances per 1,000 people	3	7
Quarterly mental health-related emergency hospital admissions per 1,000 people	15	17
Endogenous variable		
Grant amount per person in the population (£)	1.65	3.47
Controls		
Unemployment rate per individual	0.04	0.05
Income score	0.24	0.13

Income score was extracted from the 2019 English Index of Multiple Deprivation (IMD) for each LSOA. The IMD Income Score (rate) represents the proportion of the population experiencing income deprivation (i.e., receiving means-tested benefits/tax credits), and therefore ranges from 0 to 1. The mean and standard deviation reported in Table 1 are calculated for Liverpool LSOAs only

The mean number of ADQs of antidepressant prescriptions was 18,554 per 1,000 people per quarter (SD = 13,262). Other indicators of mental health service use included GP consultations (mean = 53, SD = 43), A&E attendances (mean = 3, SD = 7), and emergency hospital admissions (mean = 15, SD = 17) per 1,000 people per quarter. The grant amount per individual, used as the endogenous variable in the analysis, was on average £1.65 (SD = £3.47). Control variables included the local unemployment rate (mean = 0.04, SD = 0.05) and the deprivation income score (mean = 0.24, SD = 0.13).

To explore secular trends in the mental health index and its component indicators, Appendix 3 presents quarterly trends from 2018 to 2023 stratified by social housing quintile. While the composite index shows variation over time, the component indicators display heterogeneous patterns across the study period, including noticeable disruptions during the COVID-19 pandemic.

**Fig. 3:**
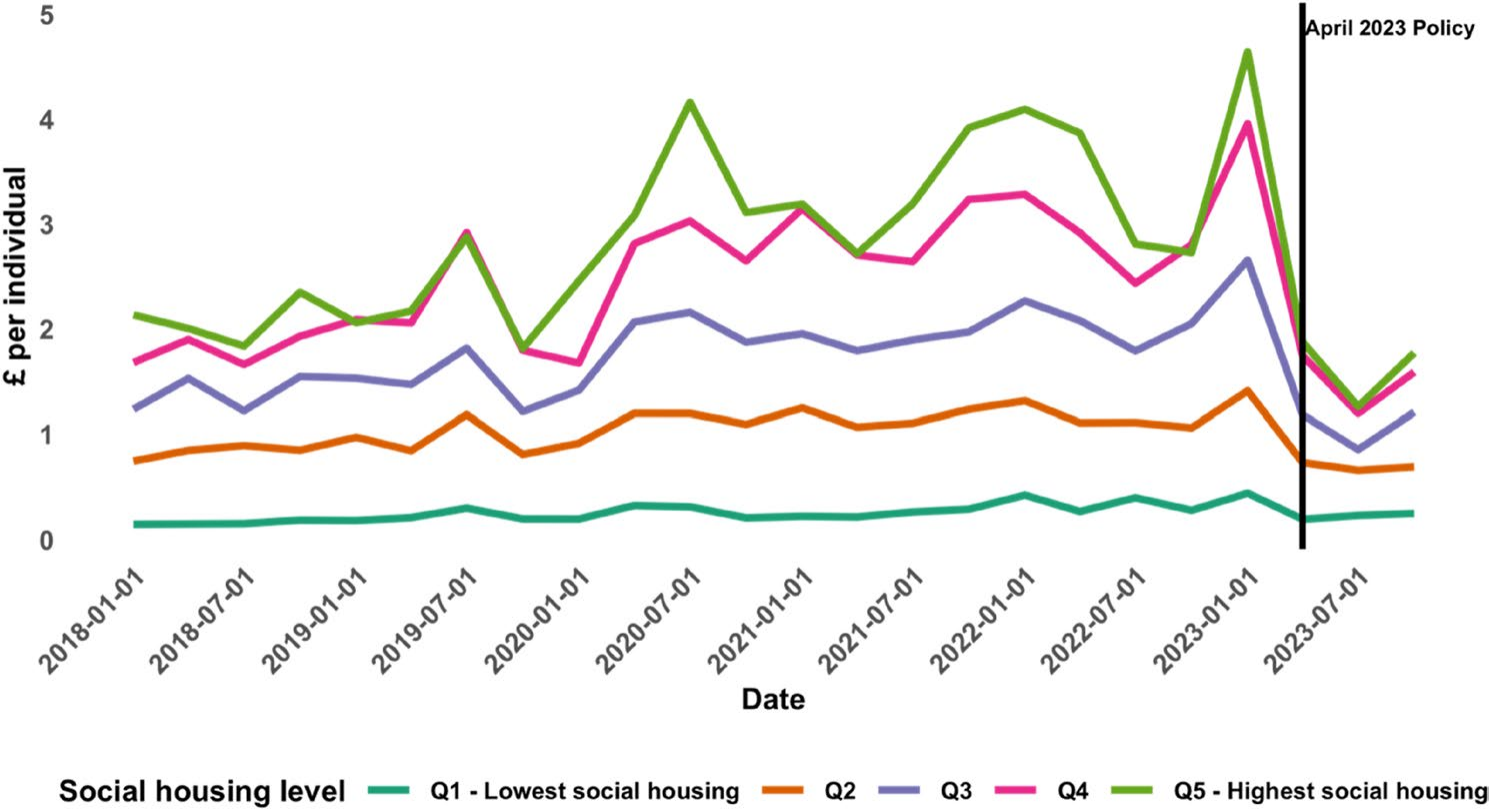
Trends in the Liverpool Citizens Support Scheme financial value of grants per person by social housing quintile between January 2018 to December 2023

Figure 3 shows the quarterly average Liverpool Citizens Support Scheme financial value of grants per person between 2018 and 2023 by quintiles of social housing concentration. A pronounced decline in grant levels is evident in areas with higher concentrations of social housing following the implementation of the April 2023 policy restriction.

Table 2 shows the estimated effects of the Liverpool Citizens Support Scheme grant rate on mental health-related service utilisation from the instrumental variable analysis. For each outcome, the table reports the point estimate along with the corresponding 95% confidence interval and p-value.

**Table 2:** Instrumental variable estimates of the impact of an additional £1 in Liverpool Citizens Support Scheme grant provision per person in the population on mental health-related service utilisation from 2018 to 2023

Outcome variable	Liverpool Citizens Support Scheme grant per person			
	Estimate	Lower CI	Upper CI	*p*-value
Quarterly points on the mental health index	−0.07	−0.11	−0.02	< 0.01
Quarterly ADQs of antidepressant prescribing per 1,000 people	−309	−536	−83	0.01
Quarterly mental health-related GP consultations per 1,000 people	−2.52	−4.88	−0.15	0.04
Quarterly mental health-related A&E attendances per 1,000 people	−0.61	−1.11	−0.11	0.02
Quarterly mental health-related emergency hospital admissions per 1,000 people	−0.78	−1.83	0.27	0.15

Appendix 4 presents event-style trends in the mental health index stratified by pre-policy grant intensity. These descriptive trends are directionally consistent with the instrumental variable estimates, although visual divergence across groups is modest

The results indicated that greater levels of Liverpool Citizens Support Scheme grant provision were consistently associated with reductions in mental health-related service utilisation. Specifically, a £1 increase in Liverpool Citizens Support Scheme grants per person in the population per quarter was associated with a 0.07-point reduction in the quarterly mental health index (95% CI: −0.11 to −0.02; *p* < 0.01), indicating lower mental health-related service utilisation. Specific outcomes also showed beneficial associations: an additional £1 in Liverpool Citizens Support Scheme grant per person per quarter was significantly associated with a reduction of 309 ADQs of antidepressants prescribed per 1,000 people per quarter (95% CI: −536 to −83; *p* = 0.01), and a reduction of 2.52 mental health-related GP consultations per 1,000 people per quarter (95% CI: −4.88 to −0.15; *p* = 0.04). In addition, a £1 increase in grant provision per person per quarter was associated with a reduction of 0.61 A&E visits per 1,000 people per quarter (*p* = 0.02, 95% CI: −1.11 to −0.11) and 0.78 emergency hospital admissions per 1,000 people per quarter, although the latter association did not reach statistical significance.

To quantify the cost savings attributable to the Liverpool Citizens Support Scheme, we multiplied the estimated effect of the Liverpool Citizens Support Scheme grant rate on each mental health-related service utilisation by the corresponding unit cost of the relevant healthcare service: antidepressant prescribing (£0.20), GP consultations (£52), A&E attendances (£414), and emergency hospital admissions (£2,431). As the estimated effects were expressed per 1,000 people, we divided them by 1,000 to calculate per person savings. Finally, the per person savings were scaled by the total value of Liverpool Citizens Support Scheme grants to estimate the overall cost savings to the healthcare system.

Based on this approach, each additional £1 in Liverpool Citizens Support Scheme grant provision per person per quarter was associated with an estimated quarterly reduction in mental health-related healthcare costs per person of approximately £0.45 (95% CI: £0.07 to £0.82), including £0.06 for antidepressant prescribing, £0.13 for GP consultations, and £0.25 for A&E attendances. The estimated saving for emergency hospital admissions was excluded from the total due to a lack of statistical significance. Between 2018 and 2023, a total of £16,432,789 was granted through the Liverpool Citizens Support Scheme, corresponding to an estimated total NHS saving of £7,394,755 (95% CI: £1,150,295 to £13,474,887) and a net intervention cost of £9,038,034 (95% CI: £2,957,902 to £15,282,494), when accounting for cost savings to the NHS from prevented healthcare utilisation. This does not account for other costs or savings that might result from increased or decreased use of other council services that are caused by the Liverpool Citizens Support Scheme intervention.

### Sensitivity analysis

#### Testing the validity of the instrumental variable

The validity of the IV approach relies on two core assumptions: relevance and exogeneity. Table 3 presents the results of tests assessing the validity of the IV approach in relation to these assumptions.

**Table 3:** Diagnostic tests for the IV estimator

First stage results	Estimate	*p*-value	F-test
Grant rate	−1.83	< 0.01	123.85

Second stage test: Wu-Hausman	Statistics	*p*-value	
Quarterly points on the mental health index	2.42	0.12	
Quarterly ADQs of antidepressant prescribing per 1,000 people	14.75	< 0.01	
Quarterly mental health-related GP consultations per 1,000 people	23.80	< 0.01	
Quarterly mental health-related A&E attendances per 1,000 people	4.54	0.03	
Quarterly mental health-related emergency hospital admissions per 1,000 people	9.51	< 0.01	

The relevance condition requires that the instrument be strongly correlated with the endogenous regressor, in this case the grant rate per individual. This assumption was formally tested using the first-stage F-statistic, which evaluates whether the instrument significantly predicts variation in the grant rate. In the first-stage regression, the coefficient on the interaction term was − 1.83 (*p*< 0.01), indicating that, after the policy was introduced, areas with higher proportions of social housing tenants received less grant provision per individual. The corresponding F-statistic was 123.85, which far exceeds the conventional threshold of 10 [23]. This result confirmed that the instrument was strongly correlated with the endogenous variable and alleviated concerns about weak instrument bias.

The exogeneity assumption requires that the instrument affects the outcome variable (i.e. mental health-related service utilisation) only through its impact on the grant rate, and not through any alternative pathways. While this assumption cannot be directly tested, we conducted the Wu–Hausman test to assess whether the grant rate itself was endogenous [24], that is, whether it was correlated with the error term in the outcome equation. Significant p-values (< 0.05) for the four mental health-related service utilisation outcomes suggested that ordinary least squares (OLS) estimates were likely biased due to endogeneity (e.g. reverse causality whereby uptake of LCSS is driven by deterioration in mental health), reinforcing the need for IV estimation. In contrast, the marginally non-significant p-value for the mental health index (*p* = 0.12) indicated that OLS and IV estimates did not differ substantially for this outcome, implying weaker endogeneity concerns. However, as tests of the underlying indicators that compose this index indicated that they were affected by endogeneity, results for the composite index should be treated with caution.

For transparency, we reported OLS fixed-effects panel estimates in Appendix Table A5.1. These models regressed quarterly mental health-related service utilisation outcomes on observed grant rates under unit and time fixed effects. As expected, OLS coefficients were generally smaller in magnitude and in some cases positive, which likely reflected reverse causality; areas with higher mental health needs tended to receive more grants. This pattern underscored the endogeneity concern highlighted by the Wu–Hausman tests and justified the use of the IV approach.

#### *Short-window analysis: 2021–2023*

The COVID-19 pandemic introduced substantial disruptions to health service utilisation patterns and welfare support delivery across the UK [25]. These disruptions may confound longer term trend estimates of the relationship between local welfare support and mental health-related service utilisation. To mitigate the influence of pandemic-related shocks, we conducted a sensitivity analysis restricted to the post-pandemic recovery period, covering quarterly data from 2021 to 2023.

Table A5.2 presents IV estimates of the quarterly impact of the Liverpool Citizens Support Scheme on mental health-related service utilisation using a restricted sample covering the period from 2021 to 2023. Compared to the main regression results, the direction of the effects remained consistent, with statistically significant associations observed for the mental health index and emergency mental health service use. Specifically, an additional £1 in Liverpool Citizens Support Scheme grant rate per person was associated with a 0.08-point reduction in the standardised mental health index (*p* < 0.01, 95% CI: −0.13 to −0.02). Furthermore, each additional £1 in Liverpool Citizens Support Scheme grants per person per quarter was associated with a reduction of approximately 0.64 A&E attendances (*p* < 0.01, 95% CI: −1.23 to −0.05) and 2.01 emergency hospital admissions (*p* < 0.01, 95% CI: −3.33 to −0.69) for mental health-related conditions per 1,000 individuals per quarter.

### Heterogeneity analysis

#### Gender

Table A5.3 in the appendix presents instrumental variable estimates of the quarterly impact of the Liverpool Citizens Support Scheme on mental health-related service utilisation by gender. Across both males and females, the estimated coefficients were consistently negative, indicating that higher Liverpool Citizens Support Scheme grant rates per individual were generally associated with reductions in mental health-related service utilisation. The magnitude of the effects was larger among males, with a statistically significant reduction of 0.11 points in the standardised mental health index, as well as decreases in A&E attendances (−1.29 per 1,000 people, *p* = 0.03) and GP consultations (−4.30 per 1,000 people, *p* = 0.06) related to mental health. Other outcomes for males, such as antidepressant prescriptions and emergency hospital admissions, also showed negative point estimates, although these did not reach statistical significance. In contrast, no statistically significant associations were observed among females across any of the five mental health-related service utilisation outcomes.

#### Age group

Table A5.4 in the appendix presents IV estimates of the quarterly impact of the Liverpool Citizens Support Scheme on mental health-related service utilisation by age groups. Across all age categories, most estimated coefficients were negative, although none were statistically significant at the 5% level. The magnitudes of the effects were relatively larger among individuals aged 65 and over compared with other groups. These findings may indicate that, unlike gender, age did not play a substantial moderating role in the relationship between Liverpool Citizens Support Scheme and mental health-related service utilisation in this context.

#### Area based income deprivation level

Table A5.5 presents the heterogeneity analysis by stratifying local areas into high- and low- groups based on the median level of income deprivation. Across both strata, the estimated effects of Liverpool Citizens Support Scheme grants per person on mental health-related service utilisation outcomes were consistently negative for most indicators; however, none of these estimates were statistically significant. These results suggested that there was no strong evidence of heterogeneous effects by local income level.

## Discussion

### Key findings

This study provides evidence consistent with the interpretation that the Liverpool Citizens Support Scheme was associated with reductions in mental health-related service utilisation. Under the assumptions of the instrumental variable design, these findings are suggestive of a causal effect; however, they should be interpreted with appropriate caution. One plausible mechanism is that the scheme may reduce acute financial stress, which could in turn influence patterns of mental health-related service utilisation. While the April 2023 policy change was leveraged as an instrument to strengthen causal inference, the results primarily reflected the impact of Liverpool Citizens Support Scheme grant provision over the entire study period rather than isolating the short-term effects of the policy change itself.

The findings of this study add evidence to a growing body of research showing that welfare provision can have significant impacts on mental health. Previous studies have primarily focused on national welfare reforms such as Universal Credit, which have been associated with worsening mental health outcomes among claimants [6, 26, 27]. In contrast, our results suggest that local welfare provision is associated with lower mental health-related service use, consistent with literature indicating that more generous or responsive welfare systems improve psychological outcomes [3, 28]. The Liverpool Citizens Support Scheme appears to mitigate financial stressors, one of the key social determinants of mental health, by providing rapid, tangible relief at moments of acute crisis. This supports theoretical frameworks that link economic security to reduced chronic stress and improved psychosocial functioning [29, 30].

Our findings resonate with recent evaluations of welfare advice delivered in primary care or community settings, which have shown reductions in anxiety and depression associated with improved financial stability [15, 16, 31]. Our evaluation of the Liverpool Citizens Support Scheme extends this evidence by demonstrating population level impacts using a quasi-experimental identification strategy; something rarely achieved in previous research on local welfare schemes. The use of an instrumental variable exploiting a policy-induced shock strengthens causal inference and helps address long-standing concerns about endogeneity and selection bias in social policy research [32, 33]. In doing so, this study contributes to a small but growing empirical literature on the health effects of local social protection systems, advancing understanding of how targeted welfare interventions can alleviate pressures on health services while promoting population mental health.

Our findings suggested that Liverpool Citizens Support Scheme welfare support had a stronger impact on males than females, consistent with previous evidence [29, 30]. This may reflect gendered differences in exposure to financial stressors and coping resources. Previous evidence indicates that men often experience greater psychological strain from economic insecurity and may benefit more from interventions that alleviate financial hardship [30]. Men are also more likely to be in employment or job-seeking roles where financial shocks have immediate consequences [34], which could amplify the mental health benefits of welfare support.

In terms of magnitude, the estimated effects observed in this study were somewhat larger than those reported for a welfare advice service, called Citizens Advice on Prescription (CAP) [16], that also operates within Liverpool. This difference is plausible given that the Liverpool Citizens Support Scheme provides direct financial assistance, which is often of greater monetary value and immediacy, than the welfare advice-based support offered through CAP, although the population-average expenditure may appear modest, as it reflects total spending averaged across all residents rather than higher-intensity support directed at a smaller number of high-need households.

Furthermore, a similar analysis to that undertaken in this study was conducted by End Furniture Poverty, who computed the wider social care and public service savings of local welfare support in Liverpool. They estimated that for every £1 invested in Liverpool City Council’s local welfare provision (which includes the Liverpool Citizens Support Scheme, Discretionary Housing Payments, and a Benefits Maximisation Service), there was a return equivalent to £9.70 through reduced demand for local authority services (e.g. social care) and £14.20 through reduced demand on broader public services, including health (NHS), criminal justice, and welfare systems [35].

### Strengths

Our analysis has some notable strengths. First, we employed data covering the entire population of Liverpool over multiple years. This provided a substantially larger and more comprehensive sample than those used in prior evaluations of local welfare support schemes [36], enhancing the generalisability and statistical power of our findings. Second, we applied an instrumental variable approach to estimate the causal effect of the programme, addressing potential endogeneity between support allocation and mental health-related service utilisation [16]. Third, we were able to estimate the cost savings to the NHS resulting from the scheme.

### Limitations

Estimating causal effects using observational data remains inherently challenging. Individuals who received Liverpool Citizens Support Scheme may have differed in unobserved ways from those who did not, introducing potential risks of confounding and selection bias. Although the instrumental variable diagnostics, including the first-stage F-statistic and the Wu–Hausman test, indicate strong instrument relevance and suggest endogeneity in naïve (OLS) models, the core IV assumption of exclusion restriction cannot be directly tested [32]. This assumption requires that the policy-induced instrument affects mental health-related service utilisation outcomes solely through its impact on grant provision and not through alternative pathways. While our modelling strategy was designed to mitigate key threats to identification, residual bias cannot be ruled out. For example, confounders between the policy instrument and mental health-related service utilisation outcomes could bias the results. These would have to be factors that were specifically associated with a deterioration in mental health after April 2023 that just affected areas with higher social housing concentration beyond any deterioration in mental health associated with deprivation.

The presence of reverse causality in naïve (OLS) models further underscores the importance of careful identification in this context. Our IV estimates should therefore be interpreted not as definitive proof of causality, but as a more credible approximation of the causal effect than conventional regression approaches in the presence of endogeneity.

Even if the core IV assumptions held, the estimated effects should be interpreted as a local average treatment effect (LATE) [33]. That is, the estimates capture the effect of grant provision among areas whose level of support changed in response to the April 2023 policy restriction. The results therefore did not necessarily represent the average effect of the scheme across all recipients or all areas, but rather the effect among those whose exposure was altered by the instrument.

Importantly, the outcomes examined in this study reflect mental health-related service utilisation rather than underlying population mental health status. Reductions in service use may reflect changes in need, but could also arise from changes in healthcare availability, access, or NHS service capacity. Although we controlled for major contemporaneous policies, including the 7% rent cap, and adjusted for time trends, we cannot fully exclude residual confounding from unobserved changes in service provision or independent policy effects. Consequently, the findings should be interpreted as associations with service use rather than definitive evidence of changes in underlying mental health prevalence.

Another key limitation of this study was its reliance on outcomes derived exclusively from electronic health records [16]. These data primarily capture healthcare utilisation rather than direct assessments of mental health status or subjective wellbeing. While it is plausible to hypothesise that improvements in mental health would translate into reduced health service use, it is also possible that interventions like the Liverpool Citizens Support Scheme may initially lead to increased utilisation. By alleviating acute social and financial pressures, the scheme may have enabled individuals to seek care for previously unmet health needs. Consequently, observed reductions in utilisation may underestimate the broader implications for health and wellbeing. The effects reported here should therefore be interpreted as a conservative estimate of its overall impact on health and wellbeing. Additionally, our reliance on a limited set of indicators means that other relevant dimensions of mental health care, such as antipsychotic prescribing and referrals to psychological therapies, psychiatry, or social prescribing, were not captured at the LSOA level. These omissions may further contribute to underestimation of the intervention’s impact, particularly for individuals with severe mental illness or complex needs who are more likely to require specialist care. Moreover, the estimates do not account for additional potential benefits such as improved wellbeing, reduced demand for social care, or increased economic participation, that could result from the intervention.

Due to the ecological design of this study, we were unable to link the Liverpool Citizens Support Scheme client data with individual health records. As the analysis was conducted at the area (LSOA) level, the findings reflect population level effects and are subject to ecological fallacy. Although we exploited within-area changes over time, we cannot conclude that individuals who received support directly experienced improvements in mental health. It remains possible that changes in area-level service utilisation reflect compositional or contextual effects rather than direct treatment effects at the individual level. Consequently, the findings should be interpreted as evidence of area-level associations consistent with a causal effect under the instrumental variable assumptions. Future research linking individual-level welfare and health data is needed to validate these relationships and reduce potential bias.

Furthermore, the use of clinical codes to measure mental health-related activity in primary care has some limitations. Coding practices can vary between GP practices and over time, which may lead to misclassification or under-recording of consultations. This could affect the precision of our estimates and should be considered when interpreting the results. Although our dataset includes three quarters of post-policy data (up to December 2023), this represents a relatively short follow-up period for assessing immediate fluctuations following the policy change. Additional data points would enable more robust interrupted time series analysis; however, this was beyond the scope of our study, which focused on estimating the overall impact of Liverpool Citizens Support Scheme grant provision over the full study period rather than short-term effects.

Finally, this study did not include an external comparison group from other local authorities. While such a study design could strengthen causal inference, we focused on Liverpool because comparable linked welfare and health data for other locations in the UK were outside the scope of our study. Furthermore, local schemes differ substantially in design and generosity, which would introduce additional heterogeneity.

### Implications

Our findings have implications for UK welfare policy. Since the abolition of national discretionary welfare funds in 2013, local authorities have increasingly borne responsibility for providing crisis support. However, local welfare schemes have faced chronic underfunding and substantial regional variation in generosity and coverage [9, 12]. The evidence presented here is consistent with the interpretation that local welfare provision may do more than address immediate material deprivation; it may also be associated with measurable public health benefits. These conclusions align with broader policy arguments for embedding public health objectives within social protection systems and contribute to the case for reinvestment in locally administered welfare [8]. Our findings are particularly relevant in the context of the new Crisis and Resilience Fund, which signals a shift from short-term crisis mitigation to resilience-building approaches [37].

Welfare support provided by the Liverpool Citizens Support Scheme is targeted at individuals facing severe financial hardship and thus the mental health benefits are likely to be concentrated among lower-income groups, which are those who often face the greatest barriers to wellbeing. This suggests that the Liverpool Citizens Support Scheme may serve not only as a mechanism for short-term financial relief, but also as a tool for reducing the mental health burden unequally distributed across socioeconomic strata and serve as a means to address health inequalities. Targeted welfare schemes such as the Liverpool Citizens Support Scheme may therefore represent a cost-effective policy lever for addressing mental health disparities and alleviating strain on healthcare services.

Local knowledge of needs and rapidly changing risks can enable local welfare schemes to be targeted effectively, enabling more “shock responsive social protection systems” [38]. Associated research to this study has demonstrated how the Liverpool Citizens Support Scheme effectively targets high-need groups, particularly for households with children and may play a critical role in safeguarding children from further poverty-related trauma and instability [39]. Our study indicates that this likely has had real mental health benefits for families. The Liverpool Citizens Support Scheme, however, relies primarily on self-referral, this may lead to some barriers to access. Further benefits that could arise through intelligence led targeting, for example using data from across council and health services to proactively target groups with high needs. With increasing pressure on council finances many local welfare schemes are at risk of being cut [40]. Our findings suggest that reductions in such support may be associated with increased demand on mental health-related healthcare services.

In the current policy landscape, characterised by fiscal constraint, welfare conditionality, and ongoing reforms to Universal Credit, programmes like the Liverpool Citizens Support Scheme offer a model of socially responsive and preventive welfare provision [41]. The observed mental health benefits and associated NHS cost savings suggest that relatively modest investments in local welfare may yield wider economic returns. As the UK government and devolved administrations revisit welfare design in light of the cost-of-living crisis, there is a strong rationale for recognising local welfare support as an integral component of mental health and strategies to reduce inequalities. Maintaining and scaling such schemes could play a crucial role in mitigating the social determinants of mental ill health and strengthening the resilience of communities most affected by economic hardship. To maximise effectiveness, equity, and long-term sustainability across diverse contexts, any future expansion should be accompanied by robust monitoring and continuous evaluation.

## Supplementary Information

Supplementary Material 1.

## Data Availability

Health records analysed in this research are held by NHS Cheshire and Merseyside, while the routinely collected Liverpool Citizens Support Scheme data are held by Liverpool City Council. The data that support the findings of this study are available from these third parties, but restrictions apply to their availability, as they were used under licence for the current study and are not publicly available. Data may be available from the corresponding author upon reasonable request and with permission from the data holders.
