# Supplementary material for: Effects of the Liverpool Citizens Support Scheme on mental health-related service utilisation: evidence from a natural experiment using instrumental variable analysis
Source: BMC Public Health. 2026 May 11;26:2024. doi: 10.1186/s12889-026-27681-x (PMC13330352; doi:10.1186/s12889-026-27681-x)
Supplement: Supplementary file 1 — Supplementary Material 1. [file 12889_2026_27681_MOESM1_ESM.docx]

**Appendices**

**Appendix 1. Codes and definition used to define each outcome**

**Anti-depressant prescriptions**

The quantity of antidepressant prescribed each month included all prescriptions within the BNF chapter 4.3. The quantity of each prescription was then converted into an Average Daily Quantity (ADQ) using a look up table giving the ADQ equivalent for each formulation (see <https://pldr.org/download/emyye/ff7/Indicator_specification_p_1_07.pdf> )

**Mental health related GP consultations**

Mental health related GP consultations were defined as any GP encounter or event, limited to a maximum of 1 per day, that included a set of mental health related SNOMED codes the SNOMED codes listed are outlined below.

Table A1.1: SNOMED concept codes to search GP records for common mental health conditions.

| cluster | SNOMED Concept | Description |
| --- | --- | --- |
| Anxiety | 402191000000101 | [X] Anxiety disorders: [other specified] or [anxiety hysteria] |
| Anxiety | 192405006 | [X]Anxiety disorder, unspecified |
| Anxiety | 450751000000102 | [X]Anxiety disorder, unspecified |
| Anxiety | 192399008 | [X]Other anxiety disorders |
| Anxiety | 468761000000105 | [X]Other anxiety disorders |
| Anxiety | 192403004 | [X]Other mixed anxiety disorders |
| Anxiety | 478661000000105 | [X]Other mixed anxiety disorders |
| Anxiety | 192397005 | [X]Other phobic anxiety disorders |
| Anxiety | 402951000000107 | [X]Other phobic anxiety disorders |
| Anxiety | 268714001 | [X]Other specified anxiety disorders |
| Anxiety | 469151000000104 | [X]Other specified anxiety disorders |
| Anxiety | 192400001 | [X]Panic disorder [episodic paroxysmal anxiety] |
| Anxiety | 416621000000108 | [X]Panic disorder [episodic paroxysmal anxiety] |
| Anxiety | 192398000 | [X]Phobic anxiety disorder, unspecified |
| Anxiety | 464911000000101 | [X]Phobic anxiety disorder, unspecified |
| Anxiety | 192393009 | [X]Phobic anxiety disorders |
| Anxiety | 472131000000109 | [X]Phobic anxiety disorders |
| Anxiety | 192610003 | [X]Separation anxiety disorder of childhood |
| Anxiety | 399651000000100 | [X]Separation anxiety disorder of childhood |
| Anxiety | 386808001 | Abnormal fear |
| Anxiety | 58963008 | Acrophobia |
| Anxiety | 192042008 | Acute post-trauma stress state |
| Anxiety | 47372000 | Adjustment disorder with anxiety |
| Anxiety | 782501005 | Adjustment disorder with mixed anxiety and depressed mood |
| Anxiety | 70691001 | Agoraphobia |
| Anxiety | 191722009 | Agoraphobia with panic attacks |
| Anxiety | 61569007 | Agoraphobia without history of panic disorder |
| Anxiety | 34938008 | Alcohol induced anxiety disorder |
| Anxiety | 82339009 | Amphetamine induced anxiety disorder |
| Anxiety | 191736004 | Anancastic neurosis |
| Anxiety | 54307006 | Animal phobia |
| Anxiety | 48694002 | Anxiety |
| Anxiety | 225644006 | Anxiety about altered body image |
| Anxiety | 247808006 | Anxiety about body function or health |
| Anxiety | 702535006 | Anxiety about breathlessness |
| Anxiety | 300895004 | Anxiety attack |
| Anxiety | 231504006 | Anxiety depression |
| Anxiety | 197480006 | Anxiety disorder |
| Anxiety | 51493001 | Anxiety disorder caused by cocaine |
| Anxiety | 724722007 | Anxiety disorder caused by dissociative drug |
| Anxiety | 2.2621E+13 | Anxiety disorder caused by drug |
| Anxiety | 724723002 | Anxiety disorder caused by ketamine |
| Anxiety | 724708007 | Anxiety disorder caused by MDMA (methylenedioxymethamphetamine) |
| Anxiety | 724654009 | Anxiety disorder caused by opioid |
| Anxiety | 55967005 | Anxiety disorder caused by phencyclidine |
| Anxiety | 762331007 | Anxiety disorder caused by stimulant |
| Anxiety | 737341006 | Anxiety disorder caused by synthetic cannabinoid |
| Anxiety | 762515000 | Anxiety disorder caused by synthetic cathinone |
| Anxiety | 52910006 | Anxiety disorder due to a general medical condition |
| Anxiety | 37868008 | Anxiety disorder of adolescence |
| Anxiety | 53467004 | Anxiety disorder of childhood |
| Anxiety | 109006 | Anxiety disorder of childhood OR adolescence |
| Anxiety | 788866004 | Anxiety due to dementia |
| Anxiety | 231506008 | Anxiety hysteria |
| Anxiety | 94641000119109 | Anxiety in pregnancy |
| Anxiety | 207363009 | Anxiety neurosis |
| Anxiety | 70655008 | Caffeine induced anxiety disorder |
| Anxiety | 34563004 | Cancer phobia |
| Anxiety | 39951001 | Cannabis induced anxiety disorder |
| Anxiety | 192611004 | Childhood phobic anxiety disorder |
| Anxiety | 191708009 | Chronic anxiety |
| Anxiety | 19887002 | Claustrophobia |
| Anxiety | 191737008 | Compulsive neurosis |
| Anxiety | 191733007 | Cyesiophobia |
| Anxiety | 38617005 | Dental phobia |
| Anxiety | 192108001 | Disturbance of anxiety and fearfulness in childhood and adolescence |
| Anxiety | 192111000 | Disturbance of anxiety and fearfulness in childhood and adolescence NOS |
| Anxiety | 657791000000107 | Disturbance of anxiety and fearfulness in childhood and adolescence NOS |
| Anxiety | 371631005 | Episodic paroxysmal anxiety disorder |
| Anxiety | 191728008 | Fear of crowded places |
| Anxiety | 102912007 | Fear of death |
| Anxiety | 21897009 | GAD - Generalised anxiety disorder |
| Anxiety | 15277004 | Hallucinogen induced anxiety disorder |
| Anxiety | 20876004 | Inhalant induced anxiety disorder |
| Anxiety | 54587008 | Isolated phobia |
| Anxiety | 70997004 | Mild anxiety |
| Anxiety | 61387006 | Moderate anxiety |
| Anxiety | 191738003 | Obsessional neurosis |
| Anxiety | 17496003 | Organic anxiety disorder |
| Anxiety | 50026000 | Organic anxiety disorder caused by psychoactive substance |
| Anxiety | 191720001 | Phobic anxiety |
| Anxiety | 386810004 | Phobic anxiety |
| Anxiety | 47505003 | Posttraumatic stress disorder |
| Anxiety | 191709001 | Recurrent anxiety |
| Anxiety | 1686006 | Sedative, hypnotic AND/OR anxiolytic-induced anxiety disorder |
| Anxiety | 126943008 | Separation anxiety |
| Anxiety | 11806006 | Separation anxiety disorder |
| Anxiety | 85061001 | Separation anxiety disorder of childhood, early onset |
| Anxiety | 80583007 | Severe anxiety (panic) |
| Anxiety | 25501002 | Social anxiety disorder |
| Anxiety | 191724005 | Social phobia, fear of eating in public |
| Anxiety | 191725006 | Social phobia, fear of public speaking |
| Anxiety | 191726007 | Social phobia, fear of public washing |
| Anxiety | 231521002 | Weight fixation |
| Anxiety symptoms | 859891000000103 | Able to manage anxiety |
| Anxiety symptoms | 247825008 | Anxiety about behaviour or performance |
| Anxiety symptoms | 225642005 | Anxiety about not coping with parenthood |
| Anxiety symptoms | 225635005 | Anxiety about treatment |
| Anxiety symptoms | 247805009 | Anxiety and fear |
| Anxiety symptoms | 69479009 | Anxiety hyperventilation |
| Anxiety symptoms | 198288003 | Anxiety state |
| Anxiety symptoms | 191711005 | Anxiety state NOS |
| Anxiety symptoms | 633361000000109 | Anxiety state NOS |
| Anxiety symptoms | 191704006 | Anxiety state unspecified |
| Anxiety symptoms | 621271000000109 | Anxiety state unspecified |
| Anxiety symptoms | 286709003 | Character trait finding of level of anxiety |
| Anxiety symptoms | 81350009 | Free-floating anxiety |
| Anxiety symptoms | 286644009 | Level of anxiety |
| Anxiety symptoms | 1149156003 | Reduced level of anxiety |
| Depression | 310495003 | [X]Mild depression |
| Depression | 430421000000104 | [X]Mild depressive episode |
| Depression | 465441000000108 | [X]Moderate depressive episode |
| Depression | 755331000000108 | [X]Recurrent major depressive episodes, severe, with psychosis, psychosis in remission |
| Depression | 397711000000100 | [X]Severe depressive episode with psychotic symptoms |
| Depression | 397701000000102 | [X]Severe depressive episode without psychotic symptoms |
| Depression | 755321000000106 | [X]Single major depressive episode, severe, with psychosis, psychosis in remission |
| Depression | 83458005 | Agitated depression |
| Depression | 788120007 | Antenatal depression |
| Depression | 790961000000101 | Antenatal depression |
| Depression | 231504006 | Anxiety depression |
| Depression | 191659001 | Atypical depressive disorder |
| Depression | 191627008 | Bipolar affective disorder, current episode depression |
| Depression | 191634005 | Bipolar affective disorder, currently depressed, in full remission |
| Depression | 192080009 | Chronic depression |
| Depression | 14183003 | Chronic major depressive disorder, single episode |
| Depression | 357705009 | Cotard syndrome |
| Depression | 35489007 | Depressed |
| Depression | 196381000000100 | Depression resolved |
| Depression | 191495003 | Depressive disorder caused by drug |
| Depression | 698957003 | Depressive disorder in remission |
| Depression | 78667006 | Depressive neurosis |
| Depression | 300706003 | Endogenous depression |
| Depression | 191608002 | Endogenous depression - recurrent |
| Depression | 274948002 | Endogenous depression - recurrent |
| Depression | 231499006 | Endogenous depression first episode |
| Depression | 321717001 | Involutional depression |
| Depression | 370143000 | Major depression |
| Depression | 63412003 | Major depression in complete remission |
| Depression | 30605009 | Major depression in partial remission |
| Depression | 42810003 | Major depression in remission |
| Depression | 70747007 | Major depression single episode, in partial remission |
| Depression | 36923009 | Major depression, single episode |
| Depression | 19527009 | Major depression, single episode, in complete remission |
| Depression | 42925002 | Major depressive disorder, single episode with atypical features |
| Depression | 69392006 | Major depressive disorder, single episode with catatonic features |
| Depression | 63778009 | Major depressive disorder, single episode with melancholic features |
| Depression | 25922000 | Major depressive disorder, single episode with postpartum onset |
| Depression | 430852001 | Major depressive disorder, single episode, severe with psychotic features |
| Depression | 231500002 | Masked depression |
| Depression | 87512008 | Mild major depression |
| Depression | 79298009 | Mild major depression, single episode |
| Depression | 237349002 | Mild postnatal depression |
| Depression | 40379007 | Mild recurrent major depression |
| Depression | 720454007 | Minimal major depression one episode |
| Depression | 310496002 | Moderate depression |
| Depression | 832007 | Moderate major depression |
| Depression | 15639000 | Moderate major depression, single episode |
| Depression | 16266831000119100 | Moderate major depressive disorder co-occurrent with anxiety single episode |
| Depression | 18818009 | Moderate recurrent major depression |
| Depression | 719593009 | Moderately severe depression |
| Depression | 720453001 | Moderately severe major depression one episode |
| Depression | 413169006 | On depression register |
| Depression | 1153575004 | Persistent depressive disorder |
| Depression | 58703003 | Postnatal depression |
| Depression | 104851000119103 | Postpartum major depression in remission |
| Depression | 231485007 | Post-schizophrenic depression |
| Depression | 426578000 | Premenstrual dysphoric disorder in remission |
| Depression | 191455000 | Presenile dementia with depression |
| Depression | 192049004 | Prolonged depressive adjustment reaction |
| Depression | 765176007 | Psychosis and severe depression co-occurrent and due to bipolar affective disorder |
| Depression | 73867007 | Psychotic depression |
| Depression | 191676002 | Psychotic reactive depression |
| Depression | 87414006 | Reactive depression |
| Depression | 288751000119101 | Reactive depressive psychosis, single episode |
| Depression | 40568001 | Recurrent brief depressive disorder |
| Depression | 191616006 | Recurrent depression |
| Depression | 1089641000000100 | Recurrent depression with current moderate episode |
| Depression | 1089511000000100 | Recurrent depression with current severe episode and psychotic features |
| Depression | 66344007 | Recurrent major depression |
| Depression | 46244001 | Recurrent major depression in complete remission |
| Depression | 33135002 | Recurrent major depression in partial remission |
| Depression | 68019004 | Recurrent major depression in remission |
| Depression | 268621008 | Recurrent major depressive episodes |
| Depression | 764691000000109 | Recurrent major depressive episodes, in partial remission |
| Depression | 764701000000109 | Recurrent major depressive episodes, in remission |
| Depression | 191610000 | Recurrent major depressive episodes, mild |
| Depression | 191611001 | Recurrent major depressive episodes, moderate |
| Depression | 764611000000100 | Recurrent major depressive episodes, severe |
| Depression | 191613003 | Recurrent major depressive episodes, severe, with psychosis |
| Depression | 413170007 | Removed from depression register |
| Depression | 247803002 | SAD - Seasonal affective disorder |
| Depression | 84760002 | Schizoaffective disorder, depressive type |
| Depression | 191459006 | Senile dementia with depression |
| Depression | 310497006 | Severe depression |
| Depression | 450714000 | Severe major depression |
| Depression | 75084000 | Severe major depression without psychotic features |
| Depression | 251000119105 | Severe major depression, single episode |
| Depression | 77911002 | Severe major depression, single episode, with psychotic features, mood-congruent |
| Depression | 20250007 | Severe major depression, single episode, with psychotic features, mood-incongruent |
| Depression | 76441001 | Severe major depression, single episode, without psychotic features |
| Depression | 237350002 | Severe postnatal depression |
| Depression | 28475009 | Severe recurrent major depression with psychotic features |
| Depression | 36474008 | Severe recurrent major depression without psychotic features |
| Depression | 764711000000106 | Single major depressive episode, in remission |
| Depression | 191601008 | Single major depressive episode, mild |
| Depression | 191604000 | Single major depressive episode, severe, with psychosis |
| Depression | 1153570009 | Treatment resistant depression |
| Depression review | 413972000 | Depression annual review |
| Depression review | 413973005 | Depression interim review |
| Depression review | 883491000000106 | Did not attend depression review |
| Depression symptoms | 871840004 | Depressive episode |
| Depression symptoms | 394924000 | Depressive symptoms |

**Mental health related A&E attendances**

A&E attendance counts are sourced from NHS datasets ECDS – Emergency Care Dataset (<https://digital.nhs.uk/data-and-information/data-collections-and-data-sets/data-sets/emergency-care-data-set-ecds>). Mental health related A&E attendances were defined as all attendances at any A&E department that included a set of SNOMED codes found in any position. The set of codes were derived from a comprehensive search of SNOMED dictionary terms. These include several thousand codes and because of the length of the list we have not included all of them here. The numbers of codes found by comprehensive search in the SNOMED catalogues are in Table A1.2:

Table A1.2: Number of SNOMED codes for every category of mental health condition searched.

| condition | Number of SNOMED codes |
| --- | --- |
| Alcohol abuse | 338 |
| Suicide and self-harm | 552 |
| Eating disorders | 79 |
| Substance abuse | 2353 |
| Other (depression, severe mental illness) | 349 |

In practice, despite the large number of codes, only few of these were used in ECDS records. In the following tables we list the codes found 2018-2023 in Cheshire and Merseyside. If codes were used in less than 10 records, we have not reported them here.

Table A1.3: SNOMED codes used to define mental health related A&E attendances.

| **SNOMED code** | **Description** |
| --- | --- |
| 25702006 | Alcohol intoxication (disorder) |
| 85561006 | Uncomplicated alcohol withdrawal (disorder) |
| 66590003 | Alcohol dependence (disorder) |
| 308742005 | Alcohol withdrawal-induced convulsion (disorder) |
| 67426006 | Toxic effect of alcohol (disorder) |
| 191480000 | Alcohol withdrawal syndrome (disorder) |
| 276853009 | Deliberate self-injury |
| 72366004 | Eating disorder |
| 56882008 | Anorexia nervosa |
| 77675002 | Anorexia nervosa, restricting type |
| 66214007 | Substance misuse |
| 1156755000 | Poisoning caused by gaseous substance (disorder) |
| 295124009 | Paracetamol overdose |
| 295830007 | Overdose of antidepressant drug (disorder) |
| 307052004 | Illicit drug use |
| 242253008 | Narcotic overdose |
| 296015009 | Sedative overdose |
| 295217003 | Non-steroidal anti-inflammatory overdose |
| 296335002 | Overdose of beta-adrenergic blocking drug (disorder) |
| 296938005 | Iron product overdose (disorder) |
| 296355001 | Overdose of calcium-channel blockers (disorder) |
| 242824002 | Intentional paracetamol overdose |
| 43302000 | Anticoagulant overdose |
| 295125005 | Accidental acetaminophen overdose |
| 69322001 | Psychotic disorder |
| 13746004 | Bipolar disorder (disorder) |
| 25702006 | Alcohol intoxication (disorder) |
| 85561006 | Uncomplicated alcohol withdrawal (disorder) |
| 66590003 | Alcohol dependence (disorder) |
| 308742005 | Alcohol withdrawal-induced convulsion (disorder) |
| 67426006 | Toxic effect of alcohol (disorder) |
| 191480000 | Alcohol withdrawal syndrome (disorder) |
| 25702006 | Alcohol intoxication (disorder) |
| 66590003 | Alcohol dependence (disorder) |
| 85561006 | Uncomplicated alcohol withdrawal (disorder) |
| 308742005 | Alcohol withdrawal-induced convulsion (disorder) |
| 191480000 | Alcohol withdrawal syndrome (disorder) |
| 67426006 | Toxic effect of alcohol (disorder) |
| 276853009 | Deliberate self-injury |
| 72366004 | Eating disorder |
| 56882008 | Anorexia nervosa |
| 77675002 | Anorexia nervosa, restricting type |
| 66214007 | Substance misuse |
| 1156755000 | Poisoning caused by gaseous substance (disorder) |
| 295124009 | Paracetamol overdose |
| 295830007 | Overdose of antidepressant drug (disorder) |
| 307052004 | Illicit drug use |
| 242253008 | Narcotic overdose |
| 296015009 | Sedative overdose |
| 295217003 | Non-steroidal anti-inflammatory overdose |
| 296335002 | Overdose of beta-adrenergic blocking drug (disorder) |
| 296938005 | Iron product overdose (disorder) |
| 296355001 | Overdose of calcium-channel blockers (disorder) |
| 242824002 | Intentional paracetamol overdose |
| 43302000 | Anticoagulant overdose |
| 295125005 | Accidental acetaminophen overdose |
| 295124009 | Paracetamol overdose |
| 295830007 | Overdose of antidepressant drug (disorder) |
| 307052004 | Illicit drug use |
| 242253008 | Narcotic overdose |
| 295217003 | Non-steroidal anti-inflammatory overdose |
| 296015009 | Sedative overdose |
| 296335002 | Overdose of beta-adrenergic blocking drug (disorder) |
| 69322001 | Psychotic disorder |
| 13746004 | Bipolar disorder (disorder) |
| 69322001 | Psychotic disorder |
| 13746004 | Bipolar disorder (disorder) |

**Mental health related emergency admissions**

The ICD-10 codes used in this work were cross checked using clinical codes list repository [1] and phenotype library [2], with background from WHO specification [3] and symptoms companion [4]. Mental health related emergency admissions were defined , using Secondary Uses Services (SUS) admitted patient care spell (APCS) tables (<https://digital.nhs.uk/services/secondary-uses-service-sus>) as any emergency [Admission_Method=2], admission including any of the following codes in any diagnostic position.

Table A1.4: ICD-10 codes used to query diagnostic fields in SUS APCS to identify clusters of mental health disorders.

| Disorder cluster | ICD-10 code | Description |
| --- | --- | --- |
| Self-harm [5,6,7] |  |  |
|  | X60-X84 | Intentional self-harm |
|  | Y10-Y34 | Injury/poisoning of indeterminate intent |
| Alcohol [5,6] |  |  |
|  | F10 | Mental and behavioural disorders due to the use of alcohol |
|  | X45 | Accidental poisoning by and exposure to alcohol |
|  | X65 | Intentional self-poisoning by and exposure to alcohol |
|  | Y15 | Poisoning by exposure to alcohol of indeterminate intent |
| Drugs and substance [5,6] |  |  |
|  | F11-F19 | Mental and behavioural disorders due to psychoactive substance use (excluding alcohol) |
|  | T36-T50 | Poisoning by drugs, medicaments and biological substances |
| Eating disorders [3,4] |  |  |
|  | F50 | Eating disorders |
|  | F98.2 | Feeding disorders of infancy and childhood |
|  | F98.3 | Pica of infancy and childhood |
| Other mental disorders [3,4] |  |  |
|  | F20-F29 | Schizophrenia, schizotypal and delusional disorders |
|  | F30-F39 | Mood [affective] disorders |
|  | F40-F48 | Neurotic, stress-related and somatoform disorders |
|  | F51-F59 | Behavioural syndromes associated with physiological disturbances and physical factors (excl. eating disorders) |
|  | F60-F69 | Disorders of adult personality and behaviour |
|  | F70-F79 | Mental retardation |
|  | F80-F89 | Disorders of psychological development |
|  | F90-F98 | Behavioural and emotional disorders with onset usually occurring in childhood and adolescence |
|  | F99 | Unspecified mental disorder |
|  | R45.8 | Other symptoms and signs involving emotional state:  Suicidal ideation (tendencies) |

**Appendix 2: Details of policy changes shown in Figure 2 that have taken place between 2018-2023**

|  | **Description** |
| --- | --- |
| **Policy shock affecting LCSS need** | |
| Policy to control expenditure: limits on awards for social tenants | To control expenditure following funding cuts, those living in social housing were advised they were no longer eligible for LCSS support and instead were encouraged to seek support from their Registered Social Landlord. |
| **Policies implemented during 2018-2023** | |
| Local Housing Allowance cap | This cap limited help for social sector tenants to the level of a private tenant in an equivalent position. |
| Increase to Benefit Cap | Benefit cap amounts were increased by 10.1%, the same percentage increase as for social security benefits linked to inflation. |
| Local Housing Allowance rates reset to 30th percentile | In response to the COVID-19 pandemic, Local Housing Allowance was returned to the 30th percentile of local rents. This helped to increase Housing Benefit award levels. |
| Reduction in social sector rents | A cash reduction of 1% per annum was introduced for 4 years, which applied to Registered Social Landlords. This affected business plans of social landlords and reduced their ability to invest in new schemes. |
| Social housing rent rise capped at 7% instead of 1% | Social housing rent rises are usually capped by the government at a maximum of Consumer Price Index plus 1%, set in September every year. As a result of rising inflation and the cost-of-living crisis, rents were instead capped at 7%. This affected business plans of social landlords and reduced their ability to invest in new schemes. |
| Test and Trace Support Payments Scheme | Introduced as there was a legal duty on all those who tested positive for COVID-19, or who were a close contact, to self-isolate. Additional LCSS support was identified to support these citizens. |
| Covid Winter Support Grant | Introduced to support those most in need with the cost of food, energy (e.g. heating, cooking, lighting), water bills and other essentials. The support provided was Local Welfare Provision and LCSS was one of the mechanisms to use this funding to support citizens in hardship. |
| Universal Credit £20 per week uplift | In response to the COVID-19 pandemic, the government introduced a temporary £20 per week increase to Universal Credit to provide additional support to low income households. |
| Household Support Fund | The government introduced the Household Support Fund to assist vulnerable households with essential costs such as food, energy, and water bills. These funds were used to deliver the LCSS. |
| Increase in maximum LCSS awards in response to COVID-19 | The standard LCSS limit of two awards in a 12-month period was increased to four. Additionally, citizens furloughed without pay were given awards over a longer duration to allow them time to claim Universal Credit. Furthermore, award levels for food and fuel were increased. |
| Expanded DHP funding | Since 2016, LCC has added funding to the government allocation for DHPs to help support residents affected by welfare reforms that impact the Housing Benefit they receive. This included DHP funding of £800m for 2016-2021 with a peak of £185m in 2017/18. |
| Cost of Living Payments | These payments were available to citizens on certain benefits. This was a government initiative and during payment cycles of this support being provided demand on LCSS reduced. |

**Appendix 3: Trends in the mental health index and its four component indicators (2018–2023), stratified by social housing quintile**


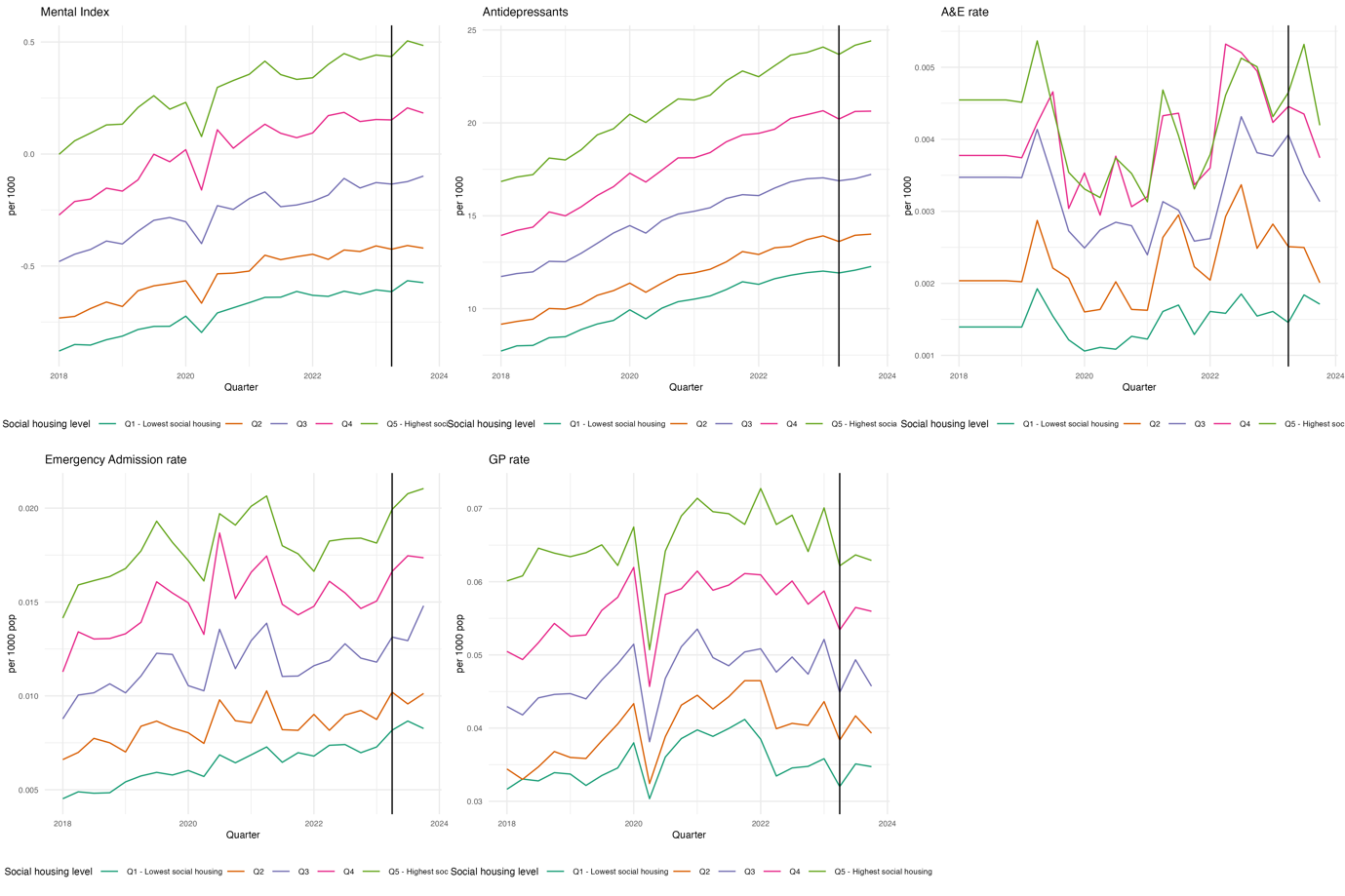


**Appendix 4: Event-style trends in the mental health-related service utilisation by pre-policy grant intensity**


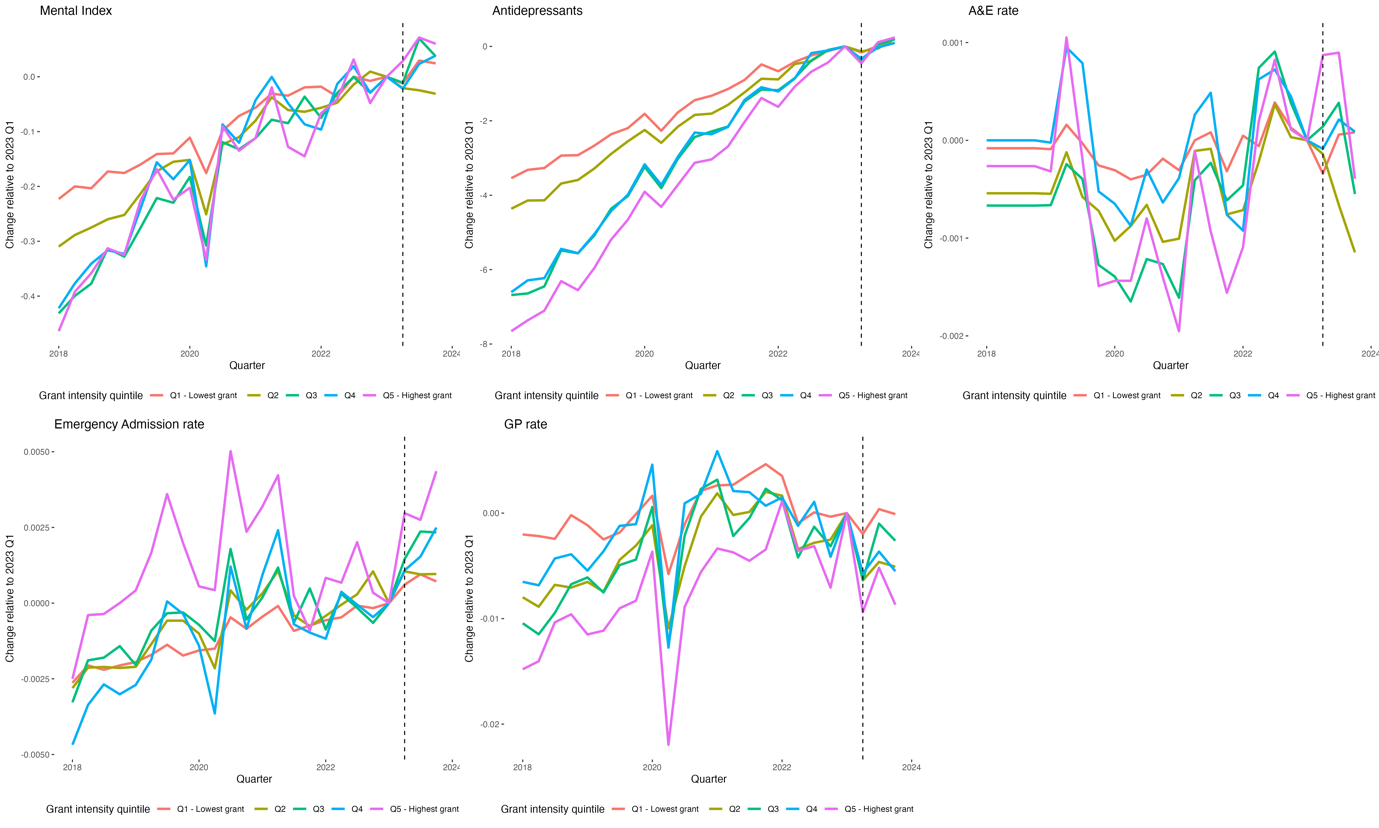


This appendix presents event-style quarterly trends for the Mental Health Index (MHI), antidepressant prescribing, A&E attendance rates, emergency admission rates, and GP consultation rates, stratified by quintiles of pre-policy grant intensity.

Pre-policy grant intensity is defined as the average per capita value of Liverpool Citizens Support Scheme grants in each area prior to April 2023. Areas were ranked according to this baseline grant rate and divided into five quintiles, where Q1 represents areas with the lowest pre-policy grant intensity and Q5 represents areas with the highest pre-policy grant intensity.

The classification is based exclusively on pre-policy data to avoid post-treatment classification bias. That is, group assignment is fixed prior to the April 2023 policy restriction and does not depend on post-policy grant changes.

The vertical dashed line indicates the implementation of the April 2023 policy restriction. All outcomes are normalised relative to their level in 2023 Q1 within each quintile to facilitate comparison of relative changes across groups. Specifically, the values shown represent:

${MHI}_{t}-\mathrm{MHI}_{2023Q1}$ (and equivalently for the other outcomes),

for each quarter t. Values below zero indicate that the outcome in that quarter was lower than its level in 2023 Q1, while values above zero indicate higher levels relative to that baseline. This normalisation is used solely to enhance visual comparability and does not imply a secular trend in levels.

Prior to the policy change, trends appear broadly parallel across quintiles for most outcomes, although there are noticeable disruptions during the COVID-19 period (2020–2021). Following the April 2023 restriction, areas in higher pre-policy grant intensity quintiles (Q4 and Q5) show directionally larger increases in the outcomes, although the visual divergence is modest.

These descriptive patterns are consistent with the marginal effects estimated in the instrumental variable framework. However, these figures present unadjusted descriptive trends and do not incorporate area or time fixed effects. As such, they should be interpreted as illustrative patterns rather than causal evidence. Formal identification of the effect relies on the instrumental variable specification described in the main text.

**Appendix 5: Sensitivity and heterogeneity analysis results**

Table A5.1: Panel regression estimates of the impact of an additional £1 in LCSS grant provision per person in the population on mental health-related service utilisation from 2021 to 2023.

| **Outcome variable** | **LCSS grant per person** | | | |
| --- | --- | --- | --- | --- |
|  | **Estimate** | **Lower CI** | **Upper CI** | **p-value** |
| Quarterly points on the mental illness index | 0.0015 | 0.0002 | 0.0028 | 0.02 |
| Quarterly ADQs of antidepressant prescribing per 1,000 people | 0.0283 | 0.0201 | 0.0364 | 0.00 |
| Quarterly mental health-related GP consultations per 1,000 people | 0.0001 | 0.0000 | 0.0002 | 0.01 |
| Quarterly mental health-related A&E attendances per 1,000 people | -0.0001 | 0.0000 | 0.0000 | 0.80 |
| Quarterly mental health-related emergency hospital admissions per 1,000 people | -0.0001 | 0.0000 | 0.0000 | 0.64 |

Note: We included unit fixed effects and time fixed effects in the model. The unemployment rate was incorporated as a control variable in the estimation.

Table A5.2: Instrumental variable estimates of the impact of an additional £1 in LCSS grant provision per person in the population on mental health-related service utilisation from 2021 to 2023.

| **Outcome variable** | **LCSS grant per person** | | | |
| --- | --- | --- | --- | --- |
|  | **Estimate** | **Lower CI** | **Upper CI** | **p-value** |
| Quarterly points on the mental illness index | -0.08 | -0.13 | -0.02 | <0.01 |
| Quarterly ADQs of antidepressant prescribing per 1,000 people | -25.66 | -249.72 | 198.39 | 0.82 |
| Quarterly mental health-related GP consultations per 1,000 people | -0.47 | -3.37 | 2.43 | 0.75 |
| Quarterly mental health-related A&E attendances per 1,000 people | -0.64 | -1.23 | -0.05 | <0.01 |
| Quarterly mental health-related emergency hospital admissions per 1,000 people | -2.01 | -3.33 | -0.69 | <0.01 |

Table A5.3: Instrumental variable estimates of the impact of an additional £1 in LCSS grant provision per person in the population on mental health-related service utilisation, disaggregated by gender.

| **Outcome variable** | **LCSS grant per person** | | | |
| --- | --- | --- | --- | --- |
|  | **Females** | | **Males** | |
|  | **Estimate** | **p-value** | **Estimate** | **p-value** |
| Quarterly points on the mental illness index | -0.02 | 0.99 | -0.11 | 0.02 |
| Quarterly ADQs of antidepressant prescribing per 1,000 people | -86.32 | 1.00 | -269.45 | 0.12 |
| Quarterly mental health-related GP consultations per 1,000 people | -3.65 | 0.98 | -4.30 | 0.06 |
| Quarterly mental health-related A&E attendances per 1,000 people | -0.33 | 0.99 | -1.29 | 0.03 |
| Quarterly mental health-related emergency hospital admissions per 1,000 people | 0.24 | 1.00 | -1.28 | 0.19 |

Table A5.4: Instrumental variable estimates of the impact of an additional £1 in LCSS grant provision per person in the population on mental health-related service utilisation, disaggregated by age group.

| **Outcome variable** | **LCSS grant per person** | | | | | | | | |
| --- | --- | --- | --- | --- | --- | --- | --- | --- | --- |
|  | **16-34** | | **35-49** | | **50-64** | | **65+** | | |
|  | **Estimate** | **p-value** | **Estimate** | **p-value** | **Estimate** | **p-value** | **Estimate** | **p-value** | |
| Quarterly points on the mental illness index | -0.11 | 1.00 | -0.01 | 1.00 | -0.04 | 0.46 | -0.65 | | 0.13 |
| Quarterly ADQs of antidepressant prescribing per 1,000 people | -894.95 | 1.00 | -323.07 | 1.00 | -12.42 | 0.96 | -1824.86 | | 0.21 |
| Quarterly mental health-related GP consultations per 1,000 people | -5.55 | 1.00 | -0.39 | 1.00 | -4.27 | 0.20 | -30.38 | | 0.14 |
| Quarterly mental health-related A&E attendances per 1,000 people | -1.36 | 1.00 | -1.01 | 1.00 | -0.35 | 0.58 | -1.24 | | 0.54 |
| Quarterly mental health-related emergency hospital admissions per 1,000 people | 0.81 | 1.00 | 0.19 | 1.00 | 0.03 | 0.98 | -13.40 | | 0.19 |

Table A5.5: Instrumental variable estimates of the impact of an additional £1 in LCSS grant provision per person in the population on mental health-related service utilisation, disaggregated by income level.

| **Outcome variable** | **LCSS grant per person** | | | |
| --- | --- | --- | --- | --- |
|  | **High-income score** | | **Low-income score** | |
|  | **Estimate** | **p-value** | **Estimate** | **p-value** |
| Quarterly points on the mental illness index | -0.12 | 0.78 | -0.01 | 0.95 |
| Quarterly ADQs of antidepressant prescribing per 1,000 people | -1911.12 | 0.70 | -1103.90 | 0.29 |
| Quarterly number of mental health-related GP consultations per 1,000 people | 5.54 | 0.80 | 3.25 | 0.68 |
| Quarterly number of mental health-related A&E attendances per 1,000 people | -2.17 | 0.74 | -0.27 | 0.86 |
| Quarterly number of mental health-related emergency hospital admissions per 1,000 people | -14.06 | 0.69 | -0.49 | 0.88 |
